# Valorization of spent disposable wooden chopstick as the CO_2_ adsorbent for a CO_2_/H_2_ mixed gas purification

**DOI:** 10.1038/s41598-022-10197-w

**Published:** 2022-04-15

**Authors:** Wanida Koo-amornpattana, Woranart Jonglertjunya, Poomiwat Phadungbut, Sakhon Ratchahat, Naphaphan Kunthakudee, Benjapon Chalermsinsuwan, Mali Hunsom

**Affiliations:** 1 Department of Chemical Engineering, Faculty of Engineering, Mahidol University, 25/25 Phuttamonthon 4 Road, Salaya, Phuttamonthon, Nakhon Pathom, 73170 Thailand; 2 Department of Chemical Technology, Faculty of Science, Chulalongkorn University, 254 Phayathai Road, Pathumwan, Bangkok, 10330 Thailand; 3 Associate Fellow of Royal Society of Thailand (AFRST), Bangkok, 10300 Thailand

**Keywords:** Energy science and technology, Engineering, Materials science

## Abstract

A series of activated carbons (ACs) derived from spent disposable wooden chopsticks was prepared via steam activation and used to separate carbon dioxide (CO_2_) from a CO_2_/hydrogen (H_2_) mixed gas at atmospheric pressure. A factorial design was employed to investigate the effects of the activation temperature and time as well as their interactions on the production yield of ACs and their CO_2_ adsorption capacity. The activation temperature exhibited a much higher impact on both the production yield and the CO_2_ adsorption capacity of ACs than the activation time. The interaction of both parameters did not significantly affect the yield of ACs, but did affect the CO_2_ adsorption capacity**.** The optimal preparation condition provided ACs with a desirable yield of around 23.18% and a CO_2_ adsorption capacity of 85.19 mg/g at 25 °C and 1 atm and consumed the total energy of 225**.**28 MJ/kg AC or 116**.**4 MJ/g-mol CO_2_**.** A H_2_ purity of greater than 96.8 mol% was achieved from a mixed gas with low CO_2_ concentration (< 20 mol%) during the first 3 min of adsorption and likewise around 90 mol% from a mixed gas with a high CO_2_ concentration (> 30 mol%) during the first 2 min. The CO_2_ adsorption on the as-prepared ACs proceeded dominantly via multilayer physical adsorption and was affected by both the surface area and micropore volume of the ACs. The adsorption capacity was diminished by around 18**%** after six adsorption/desorption cycles. The regeneration of the as-prepared chopstick-derived ACs can be easily performed via heating at a low temperature and ambient pressure, suggesting their potential application in the temperature swing adsorption process.

## Introduction

Carbon dioxide (CO_2_) is currently recognized as the most prominent contributor to global warming^1,2^. The main sectors of CO_2_ emission are energy production (~ 40%), industry (~ 23%), buildings (~ 10%), transport (~ 23%), and others (agriculture, forestry, and other land uses) (~ 5%)^3,4^. Most of the CO_2_ emissions from energy production are derived from the burning of fossil fuels, like coal (~ 72.5%) and oil and natural gas (~ 27.5%)^4^. The technology currently used to produce energy in power plants is the Integrated Gasification Combined Cycle (IGCC). With this technology, the energy carriers, such as coals, are gasified with oxygen and steam to form syngas, a mixture of carbon monoxide (CO) and hydrogen (H_2_). The obtained syngas is then further processed through the water–gas shift reaction to convert CO to CO_2_ by the reaction with H_2_O, yielding a CO_2_/H_2_ mixed gas^5–7^. To meet the target of the Paris Agreement (2015), which urged to reduce the greenhouse gas emissions by 45% by 2030 compared to 2010 and then to zero emission by 2050^8^, various strategies have been developed and applied to separate CO_2_ from a mixed gas stream. These include membrane separation, physical and chemical absorptions, cryogenic separation, and adsorption. Among these developed CO_2_ separation technologies, adsorption is the most promising and versatile technology due to its low energy consumption and operating cost, high separation efficiency, and high possibility of adsorbent regeneration^9–11^. Based on the literature, activated carbons (ACs) are one of the most appropriate adsorbents for CO_2_ capture based on their high performance and stability^12,13^.

Typically, a high CO_2_ adsorption capacity is achieved at a low temperature and high pressure^14–16^. Besides, the properties of ACs also play an important role on their CO_2_ uptake. The volume of micropores in the range of 0.33–0.82 nm of bamboo-derived ACs was the main factor responsible for the CO_2_ adsorption at 273 K and 1 bar, while neither the surface area nor the total pore volume were significant factors^13^. In contrast, both the surface area and micropore volume played a crucial role on the CO_2_ uptake by coconut shell-derived ACs^17^, local coal-derived AC activated by KOH^18^ and corncob-derived AC activated by KOH^19^. A high CO_2_ uptake of *Mesua ferrea* seed cake-derived AC was obtained when the AC had a high micropore quantity and surface functionality^20^. However, different results were observed with corn stalk-derived ACs, in which the mesopore volume played the key role in the CO_2_ adsorption capacity at a low BET surface area (< 500 m^2^/g), while the micropore area played the main role at a high BET surface area (> 500 m^2^/g)^11^. The CO_2_ uptake of water caltrop shell-derived nitrogen-doped porous carbons was enforced by the synergetic effect of N content and narrow microporous volume^21^, similar to the hazelnut shell derived N and S co-doped porous carbons, in which its CO_2_ uptake was dictated by the joint effect of narrow microporosity and N and S content^22^. The amine-impregnated AC exhibited considerably low BET surface area but importantly high CO_2_ uptake compared with the virgin AC^23^. This is because the impregnated amines acted as the active sites to adsorb the CO_2_ molecules through the chemical adsorption mechanism. The BaO-impregnated AC exhibited a considerably higher CO_2_ uptake than the unimpregnated one due to its high surface basicity^24^. The MgO-impregnated AC nanofiber can promote the CO_2_ uptake of the virgin material due to the generation of chemical bindings between the acidic CO_2_ molecules and existing basic functional groups^25^. According to above results, it seems to be that both the textural property and surface chemistry affect the quantity of the CO_2_ uptake. Nevertheless, it is still controversial to conclude which textural properties of ACs affect the CO_2_ adsorption capacity, probably due to the differences in the utilized raw materials and conditions used to prepare the ACs as well as the condition used to test the CO_2_ uptake. Nevertheless, the preparation of ACs with good textural properties and high surface might benefit for the CO_2_ adsorption.

Typically, there are two sequential steps that are involved in the production process of ACs, including carbonization and activation^26^. Carbonization (or pyrolysis) is the thermal decomposition of the raw material at high temperatures (400–1200 °C) in an inert atmosphere, such as nitrogen (N_2_) or argon (Ar), in order to eliminate volatile compounds, getting the carbonized carbonaceous material with high fixed carbon (or biochar)^12,27^. For activation, there are two established processes including physical and chemical activations. Physical activation involves thermal elimination of carbon oxides from the carbon surface using activating gases, such as CO_2_, steam, ammonia, or a combination of them^20,28,29^, while the chemical activation involves the impregnation of dehydrating agents or oxidants, such as potassium hydroxide (KOH), sodium hydroxide (NaOH), potassium carbonate (K_2_CO_3_), or zinc chloride (ZnCl_2_), and heating the mixture in an inert atmosphere^18,20,30^. Compared with physical activation, chemical activation can be achieved at a lower temperature (< 600 °C)^20^ with higher yields^26^ and higher surface areas^28^. However, it is energy consuming process due to the required severe condition to proceed the reaction^20^ and requires chemical reagents that can contaminate the obtained ACs as well as the environment^29^. Thus, physical activation is more preferable than chemical activation when considered in terms of environmental safety. The frequently used gases for physical activation are CO_2_ and steam because both gases provided ACs with a comparable BET surface area^12^. The CO_2_ activation usually facilitates the development of new micropores that are responsible for the CO_2_ adsorption^31,32^. However, it exhibits a four-fold slower reaction rate than that of steam activation^31^, leading to a long production time and high energy consumption. To conform to the need of economic feasibility, the steam activation seems more favorable than the CO_2_ activation. Nevertheless, the steam activation still faces the weakness of that the excessively high steam activation temperatures and/or times promote the creation of new micro-pores and/or widen the existing micropores, which consequently decrease the surface area and total pore volume^29,33^. Moreover, they induce a high burn-off, resulting in a low yield of ACs. Thus, knowing which preparation parameters (temperature and time) or their interaction affect the textural property of ACs and yield might help the sustainable production of ACs.

Previously, the typically precursors used to produce ACs were coal, peat, lignite, and petroleum residues^34^. However, the production of ACs from these finite resources is expensive, requires intensive regeneration, and cannot serve a high and increasing demand for global AC consumption^12^ Thus, a plethora of research have focused on the synthesis of ACs from sustainable resources, such as biomass/agricultural wastes^14,16,24,35–38^, municipal wastes^30,32,39^, and industrial wastes^40–42^. The production of AC from wastes is not only a sustainable process but also an environmentally friendly and a cost-effective strategy based on the reduction of waste disposal and the low production cost of AC^43^.

In this work, spent disposable wooden chopsticks were used as a raw material to prepare ACs by steam activation and then used to capture CO_2_ from a mixed CO_2_/H_2_ gas. A 2^k^ factorial design was carried out to investigate the effect of the activation temperature and time as well as their interactions on the yield and CO_2_ adsorption capacity of ACs. The benefit of this work is the utilization of spent disposable wooden chopsticks, one of the large scales generated municipal wastes coming from the sharp growth of food delivery services in Thailand as a sustainable carbon source to prepare ACs. This can reduce the environmental and economic burden of waste management by the government and related agencies as well as achieve the cost-effective production of ACs.

## Methods

Spent disposable wooden chopsticks were collected from an urban area in Thailand and employed as the raw material to prepare ACs. Prior to utilizing, they were cleaned, naturally dried, crushed in a knife mill and sieved to get a particle size in the range of 0.21–4.76 mm. The dry-basis proximate analysis displayed the presence of volatile matter, fixed carbon, and ash of 80.15 ± 0.38, 18.74 ± 0.37, and 1.12 ± 0.01 wt%, respectively. The ultimate analysis showed the existence of C, H, N, O, and S contents of around 54.05 ± 7.40, 6.86 ± 0.92, 0.21 ± 0.04, 38.75 ± 8.47, and 0.14 ± 0.11 wt%, respectively, and also trace minerals, such as potassium, magnesium, silicon, calcium, or iron.

### Preparation of disposable wooden chopstick-derived AC

A two-step process (carbonization and physical activation) was performed to prepare the disposable wooden chopstick-derived ACs. The carbonization was carried out at 500 °C for 15 min in a N_2_ atmosphere. In each experiment, the raw material was pre-dried at 105 °C for 3 h to eliminate the free moisture. Then, approximately 100 g of dried raw material was placed in a cylindrical stainless-steel reactor. Gaseous N_2_ (99.999%, Alternative Chem) was continuously supplied throughout the reactor at a constant flow rate of 1,000 mL/min for 30 min to build up an inert environment. Next, the reactor was slowly heated at a constant heating rate of 10 °C/min from room temperature (~ 30 °C) to 500 °C and maintained at this final temperature for 15 min. Afterwards, the reactor was left to cool down slowly to below 105 °C and the carbonized wooden material or biochar was withdrawn. For the steam activation, a 2^k^ factorial design was performed to explore the effect of the activation temperature (*A*: 700–900 °C) and activation time (*B*: 1–2 h) on the production yield and CO_2_ adsorption capacity of the obtained ACs. In each batch, approximately 40 g of the obtaining biochar was physically activated by steam in a horizontal fixed bed reactor. The steam generated from the deionized water was continuously supplied at a flow rate of 8 mL/min, while N_2_ (protecting gas) was supplied into the reactor at a rate of 1000 mL/min. After completion of the processing time, the reactor was left to cool down overnight and the resulting ACs were kept in desiccator for further characterization and utilization. Samples were coded as AC*x*–*y*, where *x* represents the activation temperature (in 100 °C units) and *y* represents the time (h). For example, AC7-1 indicates the AC which was activated at 700 °C for 1 h. The yield of ACs was computed from the weight ratio between the obtained AC and the utilized raw material as Eq. (1).

1 $$Y = \frac{{w_{AC} }}{{w_{SC} }} \times 100.$$

### Characterization

The micromorphological characteristics of the biochar and ACs were determined by scanning electron microscopy and energy dispersive X-ray spectrometry (SEM–EDX; IT-500HR JEOL) and high-resolution transmission electron microscopy (HRTEM; JEOL-JEM-3100F) with an accelerating voltage of 300 kV. The qualitative functional groups presented on the surface of all ACs were characterized by Fourier-transform infrared spectroscopy (FTIR; FT/IT-6800 JASCO). The textural properties of the ACs, including the specific surface area and pore size distribution, were computed by N_2_ adsorption/desorption isotherms at 77 K using a Multipoint Surface Area Analyzer (Micromeritics, Tristar II3020) coupled with the classical adsorption theories of Brunauer–Emmett–Teller (BET) methods.

### Adsorption capacity test

The adsorption capacity of all adsorbents was tested via the CO_2_ adsorption from a CO_2_/H_2_ mixed gas in a horizontal glass tube reactor having an inside diameter of 8 mm ID and 600 mm length at constant temperature of 25 °C and 1 atm. Prior to conducting the experiment, the AC was dried at 105 °C for 5 h to eliminate free moisture and then approximately 2 g of AC was carefully packed in the glass column, providing an effective adsorption length of around 230–250 mm. Afterwards, a CO_2_/H_2_ mixed gas was supplied at a constant flow rate of 100 mL/min into the reactor. The concentration of CO_2_ in the mixed gas stream was varied over the range of 10 to 50 mol%, controlled by mass flow controller (S48-2-HMT, Horiba). As the adsorption proceeded, the outlet gas stream was sampled to analyze the gas concentration using gas chromatography (GC; Shimadzu GC-8A) with a thermal conductivity detector (TCD) and an INJ/DET temperature of 120 °C, column temperature of 100 °C, and current of 100 mA. The amount of CO_2_ adsorption (mg CO_2_ per gram of bulk adsorbent) was obtained from integration of the transient CO_2_ concentration from the breakthrough curves using Eq. (2). The average value of at least three experimental data was reported to reduce the relative errors (3%).

2 $$q = \frac{1}{{wM_{W} }}\int\limits_{0}^{t} {\left( {C_{in} - C_{out} } \right)dt} .$$

### Modelling of adsorption isotherms

Three adsorption isotherm models were used to fit the experimental adsorption data, including Langmuir, Freundlich, and Dubinin–Radushkevich (D–R) models. The Langmuir model describes a monolayer adsorption of adsorbates onto a homogeneous surface with a constant adsorption energy in the absence of interaction between the adsorbates and neighboring sites^44,45^. The nonlinear- and linearized equations of the Langmuir model are shown in Table 1. A plot of *P*_*e*_ against *P*_*e*_/*q*_*e*_ provides the slope and *y*-intercept, which can be used to estimate *q*_*m*_ and *k*_*L*_, respectively. The Freundlich model explains a multilayer adsorption of adsorbates on the heterogeneous surface of adsorbents^46^. The adsorption energy is initially high and exponentially decreases as the degree of occupied sites increases^47,48^. Both nonlinear- and linearized forms of the Freundlich model are given in Table 1. A plot of log *P*_*e*_ versus log *q*_*e*_ allows the estimation of *n* and *k*_*F*_ from the slope and *y*-intercept, respectively. Lastly, the D–R model is appropriate to describe the equilibrium adsorption of gases and vapor on the heterogeneous surface of carbonaceous materials with a wide distribution range of pore sizes^44^. The adsorption occurs via pore volume filling rather than film formation on the perforated walls^44,49^. The original nonlinear form of the D–R model together with its linear form are also tabulated in Table 1. A plot of (ln*B*)^2^ versus ln*q*_*D*_ gives the slope and intercept, which can be used to compute the energy parameter (*E*) and *q*_*D*_, respectively.

**Table 1:** Adsorption isotherm models employed in this study^48,49^.

Isotherm model	Conventional equation	Linear equation	Plot	Slope and intercept
Langmuir	$q_{e}^{{}} =^{{}} \frac{{q_{m} k_{L} P_{e} }}{{1 + k_{L} P_{e} }}$	$\frac{{P_{e} }}{{q_{e} }}^{{}} =^{{}} \frac{1}{{k_{L} q_{m} }} + \frac{{P_{e} }}{{q_{m} }}$	*P*_*e*_ vs *P*_*e*_/*q*_*e*_	Slope = 1/*q*_*m*_ Intercept = 1/*k*_*L*_*q*_*m*_
Freundlich	$q_{e}^{{}} =^{{}} k_{F} P_{e}^{1/n}$	$\log q_{e}^{{}} =^{{}} \log k_{F} + \frac{1}{n}\log P_{e}$	log *P*_*e*_ vs log *q*_*e*_	Slope = 1/*n* Intercept = log *k*_*F*_
D–R	$q_{e}^{{}} =^{{}} q_{D} \exp \left[ { - \left( {A\ln B} \right)^{2} } \right]$*A* = *RT*/*E* and *B* = *P*_*sat*_/*P*_*e*_	$\ln q_{e}^{{}} =^{{}} \ln q_{D} - A^{2} \left( {lnB} \right)^{2}$	(ln*B*)^2^ vs ln*q*_*D*_	Slope = − *A*^2^ Intercept = ln *q*_*D*_

The goodness of fit between each isotherm model and the experimental data was determined via the determination coefficient (*R*^2^) and the normalized standard deviation (*S*), as expressed by Eqs. (3) and (4), respectively:

3 $$R^{2} = 1 - \frac{{\sum\nolimits_{i = 1}^{n} {\left( {q_{{\text{e}}} - q_{{{\text{e}},\bmod }} } \right)^{2} } }}{{\sum\nolimits_{i = 1}^{n} {\left( {q_{{\text{e}}} - \overline{q}_{{\text{e}}} } \right)^{2} } }},$$

4 $$S = \sqrt {\frac{{\left( {q_{{\text{e}}} - q_{{{\text{e}},\bmod }} } \right)/q_{{\text{e}}} }}{N - 1}} \times 100.$$

## Results and discussion

### Effect of the activation temperature and activation time

**Figure 1:**
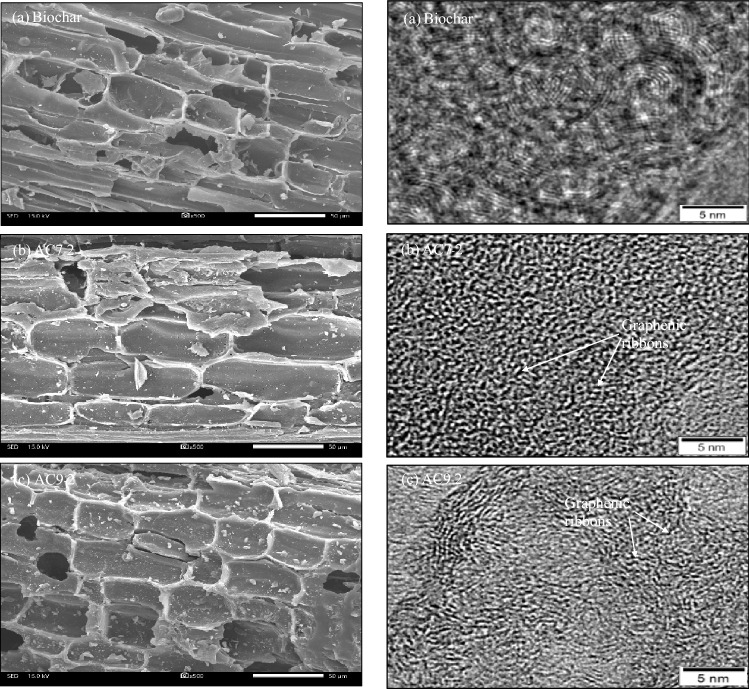
Representative SEM images (left) and HRTEM images (right) of the **(a**) biochar^50^, (**b**) AC7-2 and (**c**) AC9-2 samples.

Representative SEM and HRTEM images showing the microstructures of AC7-2 and AC9-2 together with the original biochar are illustrated in Fig. 1. The biochar showed the development of some large pores in a longitudinal direction due to the opening of vascular bundles of the wooden material during the carbonization (Fig. 1a). The HRTEM images revealed a concentric arrangement of small packets of carbon layers. Compared with the original biochar, the steam activation induced the generation of well-developed pores as well as a surface roughness due to the formation of more gasified components during the activation process. This is because the reactions between carbon and steam are endothermic, and so well-developed carbons form efficiently under elevated temperatures^33^. That is, a high temperature can effectively remove the disordered carbon coming from the deposition and decomposition of the generated tar and then facilitate the development of new pores^51^. Analysis of the AC structures by HRTEM revealed the defective graphene-like layers (dark area) of different sizes and shapes, which were bonded with the neighboring layers to create the spaces or porosity (grey area) on the surface of ACs (Fig. 1b,c).

**Figure 2:**
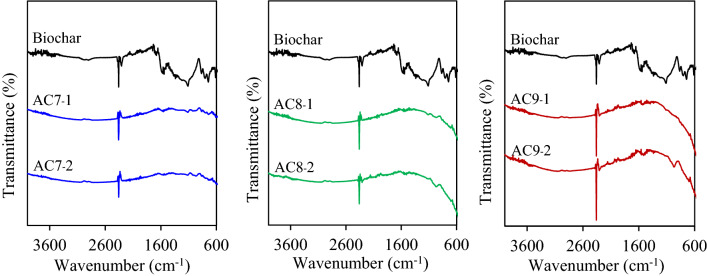
Representative FTIR spectra of the parental biochar and the six ACs prepared by steam activation.

Figure 2 shows the FTIR spectra of the parental biochar and all ACs prepared by steam activation at 700–900 °C for 1–2 h. The FTIR spectrum of the biochar that appeared at a wavenumber lower than 920 cm^−1^ indicated the presence of aromatic C–H out-of-plane bending^52^. Bands of intensity between wavenumbers of 920–1300 cm^−1^ are the overlapping C–O stretchings of various surface groups, including the C–O vibration of ethers (942–1300 cm^−1^), esters (1100–1250 cm^−1^), cyclic ethers (1140 cm^−1^), lactonic groups (1160–1370 cm^−1^), phenolic groups (1180–1220 cm^−1^), and also carboxylic acids and cyclic anhydrides (1180–1300 cm^−1^)^53^. The bands at 1480–1650 cm^−1^ indicated the presence of polyaromatic C=C stretching vibration of sp^2^ hybridized carbons^53^. The peaks found at a wavenumber of 1650–1800 cm^−1^ were attributed to the presence of the C=O stretching vibration of carboxylic and lactones. Intense spectra appeared a wavenumber of 2300–2400 cm^−1^ due to atmospheric CO_2_^54,55^. After steam activation, qualitative changes were observed in all six ACs, from which some bands were diminished. That is, the peak intensities of the aromatic C–H out-of-plane bending mode, C–O stretching vibration of different surface groups, C=C vibration of sp^2^ hybridized carbon, and C=O stretching vibration were all reduced. This was attributed to the loss of more volatile compounds that were released due to the gasification and the reaction between biochar and steam during the steam activation.

**Figure 3:**
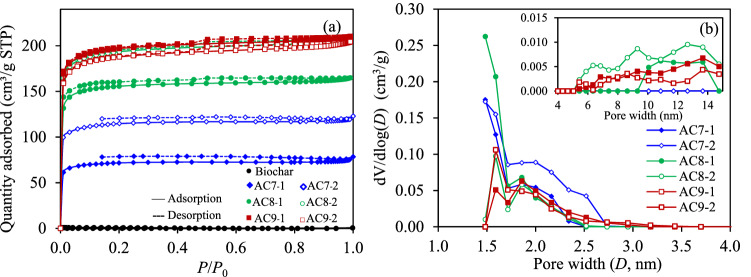
Representative (**a**) N_2_ adsorption/desorption isotherms and (**b)** pore size distribution of the parental biochar and six ACs prepared by steam activation at different activation temperatures and activation times.

The physical adsorption/desorption isotherms and pore size distribution of the ACs are shown in Fig. 3. The isotherm of AC7-1 and AC7-2 (Fig. 3a) conformed to the Type I isotherm according to the IUPAC classification^56^, indicating the presence of a predominately microporous structure with a narrow pore size distribution and a well-developed mesoporous structure^29^. The isotherms of the four ACs prepared at higher activation temperatures and times displayed a hysteresis loop; the usual characteristics of some mesopore-dominant porous materials associated from the capillary condensation in their mesopores^29^. This suggested the emergence of a mesoporous structure in the different distributions. As also displayed as inset of Fig. 3b, the generation of medium-size mesopores was initially observed for AC8-1 and was more pronounced for AC8-2, AC9-1, and AC9-2. This is attributed to the widening of the original micropores to mesopores in the presence of a high activation temperature and long activation time as well as the generation of new mesopores.

The quantitative values of the textural properties of all six ACs are tabulated in Table 2. It can be seen that, at an activation time of 1 h, all the monitored textural properties, including the *S*_BET_, *S*_t-plot_, *V*_mic_ and *V*_mes_, increased as the activation temperature increased from 700 to 900 °C. The AC8-1 exhibited the highest micropore volume ratio (*V*_mic_/*V*_mic_ + *V*_mes_) of 81.57%. At an activation time of 2 h, the *S*_BET_, *S*_t-plot_, and *V*_mic_ also increased as the activation temperature increased from 700 to 800 °C but then slightly decreased at 900 °C. This was not the case for *V*_mes_, in which it continuously increased over the whole range of activation temperatures. The micropore volume ratio decreased slightly from 79.83 to 77.18%, indicating the relatively low existence of a microporous structure at a high activation temperature and long activation time.

**Table 2:** Textural property and CO_2_ adsorption capacity at 25 °C and 1 atm of the six ACs and the parental biochar.

Sample	*S*_BET_ (m^2^/g)	*S*_t-plot_ (m^2^/g)	*V*_mic_ (cm^3^/g)	*V*_mes_ (cm^3^/g)	*V*_mic_/*V*_mic_ + *V*_mes_ (%)	*q* (mg/g)
Biochar	0.12					19.20
AC7-1	203.1	165.5	0.0944	0.0239	79.80	74.46
AC7-2	330.0	263.9	0.1484	0.0375	79.83	85.19
AC8-1	441.8	366.2	0.2076	0.0469	81.57	86.80
AC8-2	551.6	440.5	0.2505	0.0687	78.48	87.58
AC9-1	561.1	450.2	0.2558	0.0672	79.20	89.85
AC9-2	536.6	424.2	0.2412	0.0713	77.18	81.13

The CO_2_ adsorption capacity of all six ACs prepared by steam activation from a CO_2_/H_2_ mixed gas is also summarized in Table 2. The steam activation significantly improved the adsorption capacity of the biochar from around 19.20 mg/g to greater than 74.46 mg/g, a greater than 3.88-fold improvement**.** Increasing the activation time from 1 to 2 h increased the CO_2_ adsorption capacity of the ACs prepared at 700 °C, but a longer activation time at 800 °C was not significant. However, it negatively affected the CO_2_ adsorption capacity of ACs prepared at 900 °C. The AC9-1 exhibited the maximum adsorption capacity (around 89.85 mg/g), while AC9-2 displayed a remarkably lower CO_2_ adsorption capacity (81.13 mg/g).

**Figure 4:**
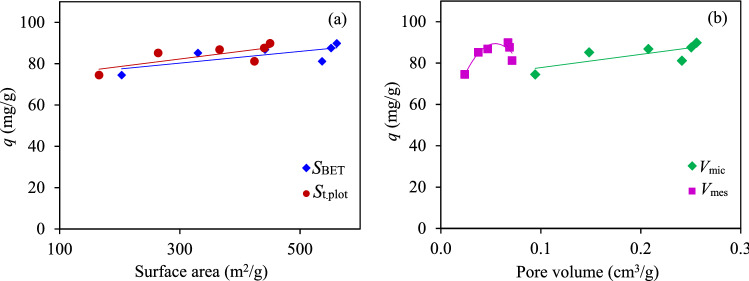
Effect of the (**a**) surface area and (**b**) pore volume of the obtained ACs on the CO_2_ adsorption capacity at 25 °C and 1 atm.

The relationship between the CO_2_ adsorption capacity and textural properties of ACs prepared at different activation temperatures and activation times is plotted in Fig. 4. The CO_2_ adsorption exhibited a direct relationship to the *S*_BET_, *S*_t-plot_, and *V*_mic_ of the ACs and a relatively fluctuating trend with respect to the *V*_mes_. This suggested that both the surface area and micropore volume were the dominant factors promoting the CO_2_ adsorption, in accord with previous studies that mentioned that micropores play a crucial rule in CO_2_ adsorption^11,57^. This is because a large quantity of CO_2_ molecules can diffuse throughout a high surface area of ACs and strongly adsorb at their micropores via van der Waal’s forces^24,58^.

In comparison, the CO_2_ uptake of the ACs prepared from various types of biomass-waste by physical activation reported in the literature are displayed in Table 3. The adsorption capacity of the chopstick-derived AC (AC7-2) was on par with those reported in the literature. The difference in adsorption capacity might be due to the differences in biomass-waste type and properties, the condition used to prepare the AC and to test the adsorption capacity (ex. gas composition and gas flow rate), as well as the equipment or reactors used to test the adsorption capacity.

**Table 3:** Comparison of the CO_2_ uptake by biomass-waste derived ACs prepared by physical activation.

Type of biomass-waste	Preparation method	Adsorption conditions	Reactor type	CO_2_ adsorption (mg/g)
Spent chopstick (this work)	Carbonization + steam activation	25 °C, 1 atm, 50% CO_2_	Fixed bed	85.19
Olive stones^32^	Carbonization + CO_2_ activation	25 °C, 1 atm, pure CO_2_	TGA analyzer	105.6
Almond shells^39^	Carbonization + CO_2_ activation	25 °C, 1 atm, 15% CO_2_	TGA analyzer	114.4
Almond shells/olive stones^30^	Single-step CO_2_ activation	30 °C, 1.18 atm, 14% CO_2_	TGA analyzer	40.48/25.52
Coconut shell^36^	Single-step CO_2_ activation	25 °C, 1 atm, pure CO_2_	TGA analyzer	78.79
Rapeseed oil cake/walnut shells mixture^59^	Carbonization + CO_2_ activation	25 °C, pure CO_2_	TGA analyzer	68.75
*Mesua ferrea* L. seed cake^20^	Hydrothermal + calcination	25 °C, 1 atm, 10% CO_2_	Fixed bed	114.4
Whitewood^60^	Carbonization + steam activation	25 °C, 1 atm, 10% CO_2_	Fixed bed	35.0
*Phoenix dactylifera* seeds^37^	Carbonization + CO_2_ activation	20 °C, pure CO_2_	Micro reaction calorimeter	141.1
Graphene oxide^61^	CO_2_ activation	25 °C, 0.9869 atm, pure CO_2_	TGA analyzer	27.2
Date stones^16^	Carbonization + CO_2_ activation	25 °C, 1.28 atm, 5% CO_2_	Fixed bed	74.8

### Optimization of the AC preparation condition

To further understand the impact of the AC preparation condition, in terms of the activation temperature (*A*) and activation time (*B*), on the production yield (*Y*) and CO_2_ adsorption capacity (*q*) of ACs, a collection of statistical models and associated estimation procedures known as analysis of variance (ANOVA) was performed. Table 4 tabulates each experimental condition in terms of coded variables and response values. It can be seen that case 1 exhibited the highest production yield, but the lowest CO_2_ adsorption, whilst case 5 showed the maximum adsorption capacity with an extremely low production yield but still in the acceptable range of the dry biomass-derived ACs, of 5–40 wt%^53^, while the lowest production yield was obtained at case 6**.** Table 5 illustrates the ANOVA analysis of these two response variables. The manipulated variables that had a *p*-value of less than 0.05 were considered as a statistically significant effect at the 95% confidential interval level. Conspicuously, the activation temperature played an important role on the production yield of ACs, while the interaction between the activation temperature and the activation time exhibited an important effect on the CO_2_ adsorption capacity.

**Table 4:** Experimental condition and response values.

Case	Sample	*A*	*B*	*Y* (%)	*q* (mg/g)	Variables	(− 1)	(+ 1)
1	AC7-1	− 1	− 1	24.31	74.46	*A*: Activation temperature (°C)	700	900
2	AC7-2	− 1	1	23.18	85.19			
3	AC8-1	0	− 1	17.04	86.80	*B*: Activation time (h)	1	2
4	AC8-2	0	1	8.08	87.58			
5	AC9-1	1	− 1	6.51	89.85			
6	AC9-2	1	1	1.57	81.13

**Table 5:** ANOVA analysis of the response variables.

Response variable	Source of variation	Sum square	Degree of freedom	Mean square	*F*-value	*p*-value
*Y*	*A*	388.28	1	388.2	65.76	< 0.01
	*B*	37.66	1	37.66	6.38	0.09
	Residual	17.71	3	5.900		
	Cor total	443.65	5			
*q*	*A*	32.02	1	32.02	3.34	0.16
	*AB*	94.57	1	94.57	9.87	0.05
	Residual	28.74	3	9.58		
	Cor total	155.3	5			

**Figure 5:**
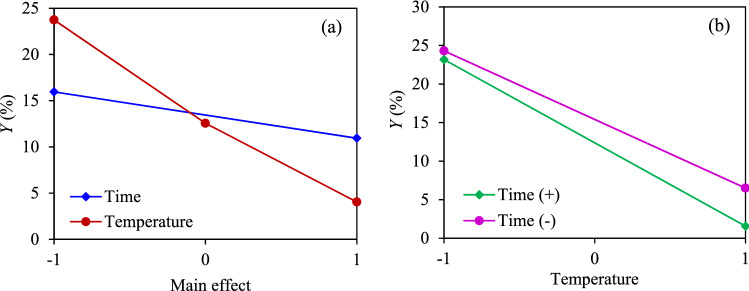
Plots of the (**a**) main effect and (**b**) interaction effect on the AC production yield.

Plots of the main and interaction effects of the manipulated variables on the production yield of ACs are shown in Fig. 5. Both a high activation temperature and long activation time exhibited a negative effect on the AC production yield. The activation temperature exhibited a much steeper plot than that of the activation time, indicating its greater impact on the AC production yield than the activation time (Fig. 5a), while the interaction effect of both manipulated variables was not pronounced in this study range, as can be seen by the parallel graph lines (Fig. 5b). This is because the steam activation induced the decomposition of cellulose and hemicellulose leading to the formation of a high porosity in the structure of AC, which allowed the diffusion of the oxidizing agent into the carbon structures and consequently reacted with the lignin^62,63^. Upon increasing the temperature and time, more volatiles were released due to the gasification and the reaction between biochar (C_f_) and steam, according to reactions (R1) and (R2)^64^, thus resulting in the decreasing yield of ACs^65^.

R1 $${\text{C}}_{{\text{f}}} + {\text{H}}_{{2}} {\text{O}} \to {\text{CO}}_{{2}} + {\text{2H}}_{{2}} ,$$

R2 $${\text{C}}_{{\text{f}}} + {\text{H}}_{{2}} {\text{O}} \to {\text{C}}({\text{O}}) \, + {\text{H}}_{{2}} .$$

**Figure 6:**
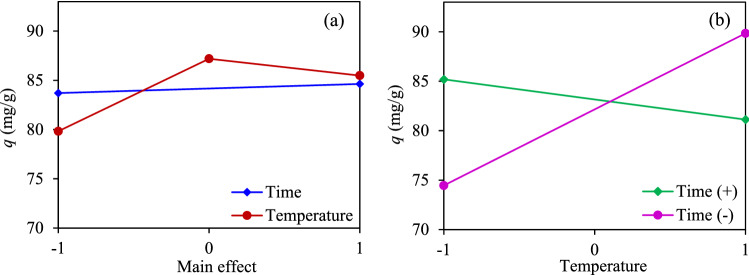
Plots of the (**a**) main and (**b**) interaction effects on the CO_2_ adsorption.

Figure 6 depicts the plots of the main and interaction effects for both manipulated variables on the CO_2_ adsorption capacity. Both the activation temperature and time exhibited a slight impact on the CO_2_ adsorption capacity. The maximum adsorption was observed at a suitable activation temperature (Fig. 6a), indicating a non-linear relationship between the activation temperature and CO_2_ adsorption capacity. According to the interaction effect plot (Fig. 6b), the intersect of the two linear-curves was observed, indicting a significant interaction effect between the activation temperature and activation time on the CO_2_ adsorption capacity. A high CO_2_ adsorption was achieved for the ACs prepared at a lower activation temperature and a longer activation time (700 °C, 2 h), or those prepared at a high activation temperature and short activation time (900 °C, 1 h). The AC prepared at an elevated activation temperature and long activation time exhibited a markedly low CO_2_ adsorption capacity (ex. case 6), because a long activation time at an elevated temperature can induce a high degree of widening of the existing pores instead of pore-deepening and/or new pore generation^33,66,67^. This pore-widening effect was experimentally confirmed by the decreased micropore volume ratio from 79.20 to 77.18% as the reaction time increased from 1 to 2 h at 900 °C (Table 2). Based on the statistical analysis, the regression models used to predict the production yield and adsorption capacity can be written as Eqs. (5) and (6), respectively.

5 $$Y = 13.446039 - 9.852355A - 2.505472B,$$

6 $$q = 84.168498 + 2.8292162A - 4.862424AB,$$

where *A* and *B* are the coded activation temperature and time, respectively.

**Figure 7:**
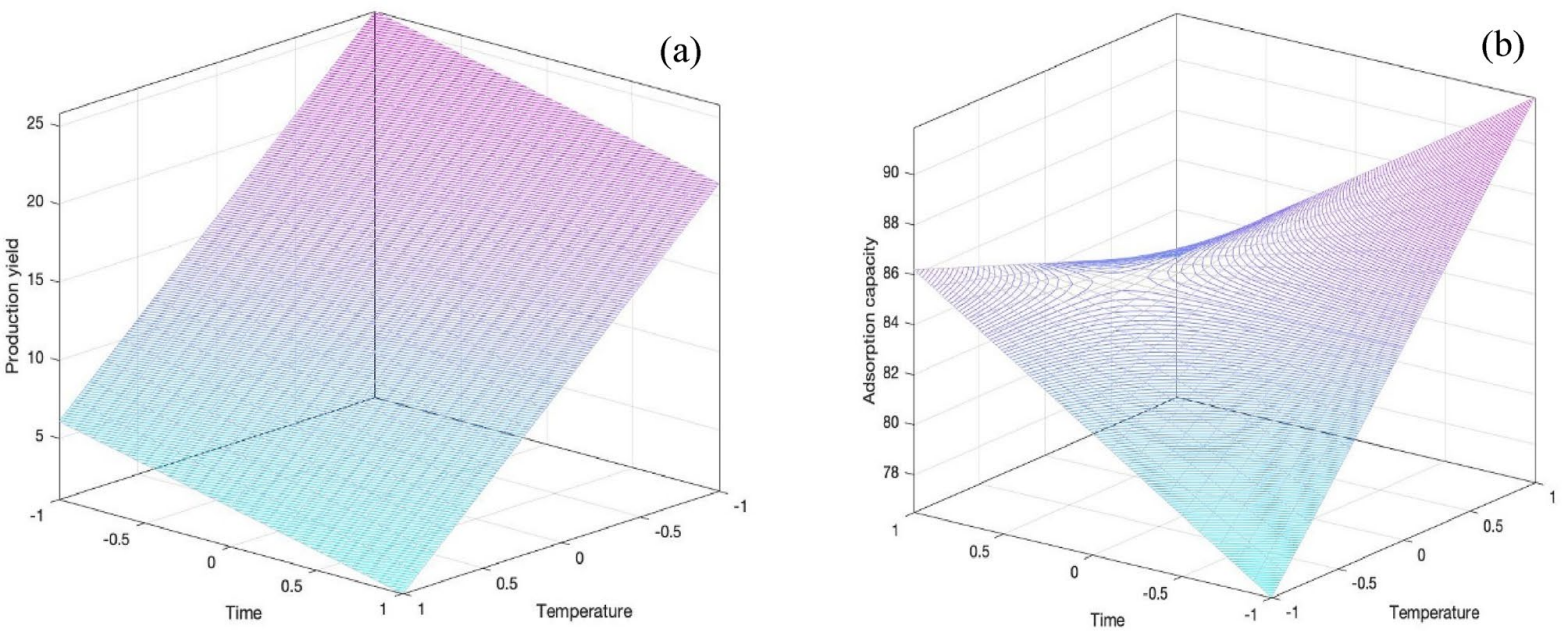
Contour plots of the (**a**) AC yield and (**b**) CO_2_ adsorption as a function of the activation temperatures and activation times.

Figure 7 shows the contour plots of both manipulated variables against both response variables**.** A high AC production yield was obtained at a low activation temperature and short activation time (Fig. 7a), while a high CO_2_ adsorption was achieved at a high activation temperature and short activation time (Fig. 7b). From an economical point of view and adsorption performance, the optimal activation temperature and activation time for the preparation of AC was found to be at 700 °C for 2 h, respectively. At this predicted condition, the approximated values of production yield and CO_2_ adsorption capacity were around 20.79 and 86.20 mg/g, respectively, which were closed to those obtained from the bench-scale experiment.

**Figure 8:**
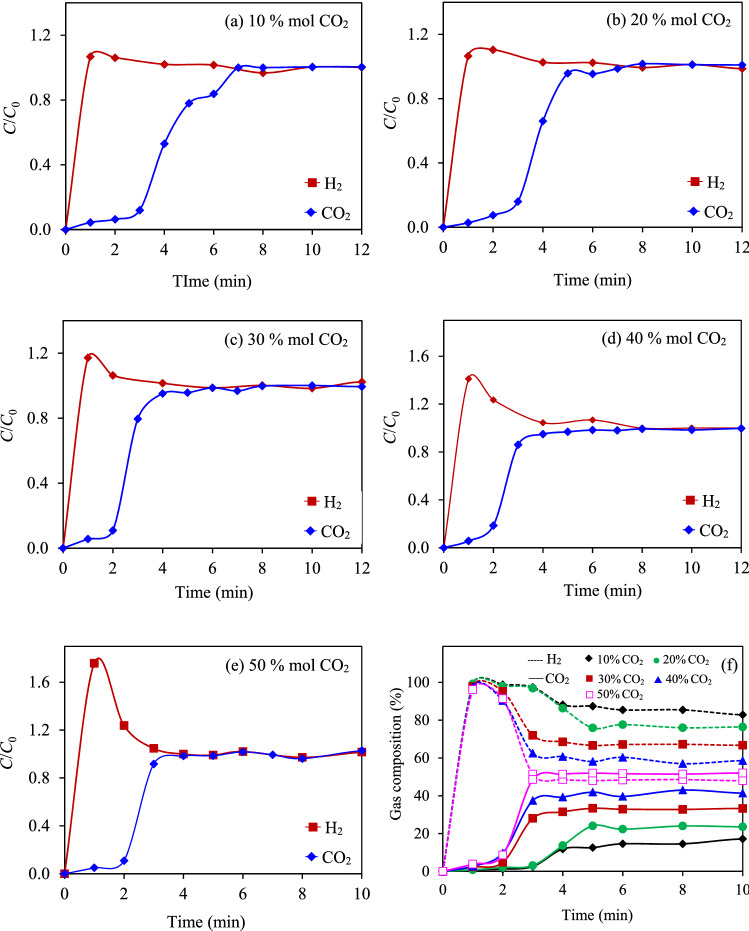
(**a**–**e**) Breakthrough curve of an equimolar CO_2_/H_2_ mixture at 25 °C and 1 atm and (**f**) the exit gas concentration profile at different inlet CO_2_ concentrations over the range of 10–50 mol% of the AC7-2.

Figure 8a–e shows the breakthrough curve of an equimolar CO_2_/H_2_ mixture at 25 °C and 1 atm and the concentration profile of the exit gas stream at different inlet CO_2_ concentrations of all the adsorbents**.** The breakthrough curve exhibited a huge roll-up of H_2_ (*C*/*C*_0_ > 1) at the early adsorption period (< 2 min). This indicted a fast exit of H_2_ in the exhaust stream or its lower adsorption compared with CO_2_^68,69^. In other words, the as-prepared AC7-2 exhibited a strong CO_2_ adsorption and a weak H_2_ adsorption. This roll-up behavior was observed over the entire investigated range of CO_2_ concentrations (10–50 mol%; data not shown), supporting that the separation of CO_2_ from H_2_ was due to thermodynamic separation^70,71^. This is because the weaker adsorbed H_2_ exhibited a fast diffusion in the porous structure of AC compared with a stronger adsorbed CO_2_, leading to a transient H_2_ rich adsorbed phase^71^. Besides, as CO_2_ is heavier than H_2_ it exhibited a higher adsorption affinity towards the AC adsorbents^9,72^, which has previously been ranked in the order of CO_2_ >  > CH_4_ > CO >  > H_2_^72^. Due to the low adsorption affinity of H_2_ from the CO_2_/H_2_ mixed gas via the AC7-2, the calculation of either the CO_2_ or H_2_ selectivity using the thermodynamic analysis by Ideal adsorbed solution theory (IAST) is not applicable^71,73^. Thus, the composition of the outlet gas stream (*M*) or the exit gas concentration profile was calculated according to Eqs. (7) and (8) and depicted in Fig. 8f.

7 $$M_{{{\text{H}}_{2} }} = \frac{{m_{{{\text{H}}_{2} }} }}{{m_{{{\text{H}}_{2} }} + m_{{{\text{CO}}_{2} }} }} \times 100,$$

8 $$M_{{{\text{CO}}_{2} }} = 100 - M_{{{\text{H}}_{2} }} .$$

According to the plot, the obtained profiles can be categorized into two distinct regions; (i) the breakthrough of H_2_ at the early adsorption period and the production of a H_2_-rich gas stream, and (ii) the breakthrough of CO_2_ and transient to reach the feed composition. The gas stream with the high CO_2_ concentration exhibited a breakthrough faster than that with the low CO_2_ concentration. The mixed gas stream with a low CO_2_ concentration (< 20 mol%) gave an exit stream with a high H_2_ purity (> 96.8 mol%) during the first 3 min of adsorption and then lessened afterwards to reach the feed composition, while the mixed gas stream with a CO_2_ concentration of more than 30 mol% provided an exit gas stream with a H_2_ purity greater than 90% during the first 2 min of adsorption time. This information will help engineers to design an industrial scale CO_2_ capture system using the adsorption-based separation process from a CO_2_/H_2_ mixed gas over a wide range of CO_2_ concentrations.

### Adsorption isotherm

**Figure 9:**
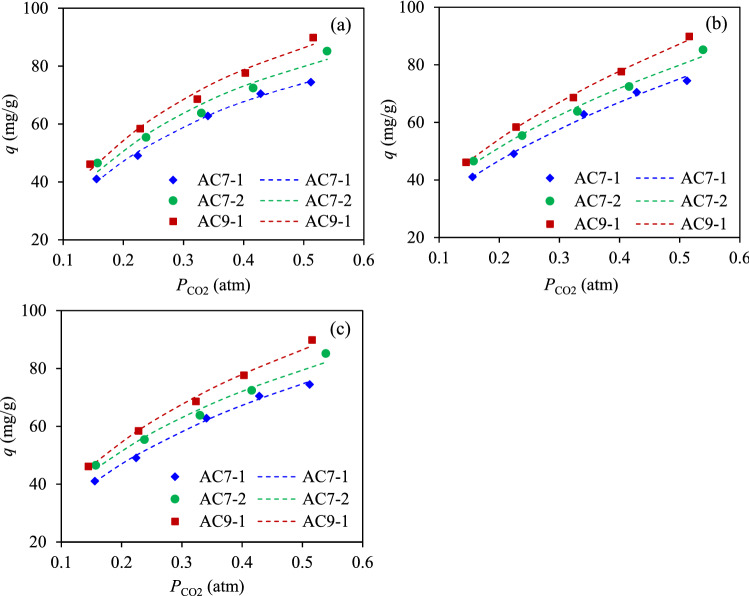
Adsorption isotherms of CO_2_ on three ACs fitted by the (**a**) Langmuir, (**b**) Freundlich, and (**c**) D–R models.

Figure 9 depicts the fitting curves between the experimental data (marker point) and isotherm results (dashed line) for the CO_2_ adsorption by three ACs at 25 °C and 1 atm in the presence of different CO_2_ concentrations. The obtained coefficients and fitting quality were considered in terms of the determination coefficient (*R*^2^) and the normalized standard deviation (*S*), with the results summarized in Table 6.

**Table 6:** Obtained constants for different adsorption isotherm models.

		AC7-1	AC7-2	AC9-1
Langmuir	*q*_*m*_ (mg/g)	119.80	129.93	142.15
	*k*_*L*_ (atm^−1^)	3.24	3.21	3.10
	*R*^2^	0.9945	0.9670	0.9805
	*S* (%)	1.84	4.56	3.33
Freundlich	*k*_*F*_ (mg/g-atm^1/n^)	107.6	111.9	125.0
	*n*	1.9324	2.0663	1.9298
	*R*^2^	0.9954	0.9917	0.9983
	*S* (%)	1.72	2.13	1.06
D–R	*q*_*D*_ (mg/g)	229	227	263
	*E* (kJ/mol)	11.3	11.7	11.4
	*R*^2^	0.9965	0.9845	0.9952
	*S* **(%)**	1.50	2.91	1.06

The adsorption capacity increased as the increased CO_2_ concentration, which was attributed to the high driving force of the CO_2_ concentration between the bulk phase and the surface of AC that can promote a high mass transfer rate^74,75^. For all the explored ACs, the Freundlich model provided a better fit with the experimental results over the entire range of CO_2_ concentrations than the Langmuir model, considered in terms of the higher *R*^*2*^ and *S* values. This suggests that the CO_2_ adsorption on the spent chopstick-derived AC occurred predominantly via a multilayer adsorption with a heterogeneous surface binding^44,49^. The value of *n* was higher than 1 (*n* > 1), confirming a favorable adsorption^75^ as well as its high degree of heterogeneity and good adsorption intensity^49,76^. The adsorption energy parameters **(***E***)** obtained from the D-R isotherm model varied in the range of 11.3 to 11.7 kJ/mol, which were between 8 and 20 kJ/mol, and so were neither purely physical adsorption (< 8 kJ/mol) nor chemical adsorption (> 20 kJ/mol)^77^. Nevertheless, a deviation of energy parameters from a value of 8 kJ/mol of around 25% indicated a predominately physical adsorption. In other words, the CO_2_ molecules were dominantly adsorbed via the intermolecular cohesion forces at the pore surface and small part of them were adsorbed via the surface functionalities that originated from pyrolysis as well as inorganic matters^20^.

**Figure 10:**
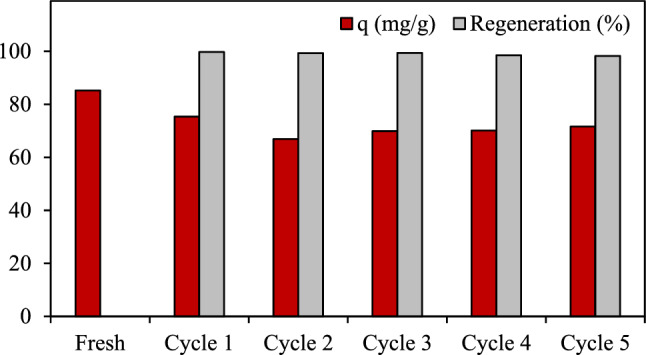
The CO_2_ adsorption capacity at 25 °C and 1 atm of the AC7-2 over six successive adsorption/desorption cycles.

The recyclability of an adsorbent plays an essential role in the economics of a commercial scale operation, where a high cyclic stability of any employed adsorbent is required. In this work, the cyclic stability of the AC7-2 sample was tested by repetitive adsorption of CO_2_ from a CO_2_/H_2_ mixed gas (50 mol% CO_2_) at 25 °C and 1 atm. After each particular adsorption, the adsorbed CO_2_ on the surface of the AC7-2 was simply desorbed in air at 105 °C at ambient pressure and then subjected to re-adsorb CO_2_ from the mixed gas stream at the same CO_2_ concentration. As shown in Fig. 10, the fresh AC exhibited a CO_2_ adsorption capacity of 85.19 mg/g and this dropped slightly with increasing regenerative cycles. This suggested that the regeneration of AC via the heating process is effective to remove the weak adsorbed CO_2_ at the outer layer of a multi-layer adsorption. At the even after six adsorption/desorption cycles, the employed adsorbent depicted a minimal loss of CO_2_ adsorption capacity of around 18% of its initial capacity without any significant loss of ACs. Based on the regeneration results, the as-prepared chopstick-derived AC7-2 is recommended as the candidate adsorbent for CO_2_ adsorption in the temperature swing adsorption process in air at low temperature (ex. 105 °C) and ambient pressure. Otherwise, to enhance a higher recyclability, other regeneration procedures such as depressurization or chemical regeneration should be investigated^78^.

### Energy consumption analysis

The analysis of required energy to produce the spent disposable wooden chopstick-derived AC by steam activation was adopted from the energy model of Maski et al.^79^ and Chen et al.^80^. The energy demand in the production process was separated into two sub-production processes, including the carbonization for biochar production and the steam activation. To perform the energy balance, the heat capacitis of chopstick (*C*_*p*,*sc*_) and biochar (*C*_*p*,char_) were estimated from Eqs. (9) and (10), respectively^81^.

9 $$C_{p,sc} = 1500 + T,$$

10 $$C_{p,char} = 420 + 2.09T - 6.85 \times 10^{ - 4} T^{2} .$$

For the basis of 1 kg of chopstick with 7.73 wt% moisture content, the energy consumption used to produce the chopstick-derived AC was determined as shown below.

For the carbonization, there are two types of energy required to prepare biochar; (i) energy required to dry the moisture-bearing chopstick and (ii) energy required to heat chopstick (*E*_*CS*_) from room temperature (30 °C) to 500 °C and holding at this temperature for 15 min.

(i) Energy required to dry the moisture-bearing chopstick was the summation of energy required to raise the temperature of moisture from 30 to 100 °C (*E*_*w*_) and energy required to vaporize the moisture at 100 °C (*E*_*vap*_) as expressed by Eqs. (11) and (12), respectively.
  11 $$E_{w} = w_{m} C_{p,w} \Delta T = 0.0228\,{\text{MJ}}.$$
  12 $$E_{vap} = w_{m} \Delta H_{vap} = 0.1745\,{\text{MJ}}$$
(ii) Energy required to heat chopstick was composed of the energy required to heat chopstick from 30 to 105 °C (*E*_*cs*,heat-1_), energy required for holding chopstick at 105 °C for 3 h (*E*_*cs*,hold-1_), energy required to heat chopstick from 105 to 500 °C (*E*_*CS*,heat-2_) and energy required for holding chopstick at 500 °C for 15 min (*E*_*sc,hold*-2_) The values of *E*_*cs*,heat-1_ and *E*_*cs*,heat-2_ were estimated by Eqs. (13) and (14), respectively. Due to the complexity of reaction pathways during drying and carbonization, the energy consumptions during both periods were computed from the voltage–current–time profile of the oven and furnace, which were 0.0009 and 0.0011 MW, respectively. Based on the utilized processing time, the values of *E*_*cs*,hold-1_ and *E*_*cs*,hold-2_ were 9.979 and 0.993 MJ, respectively. Therefore, the total energy required to produce biochar per kg of chopstick was 11.7977 MJ.
  13 $$E_{sc,heat - 1} = w_{sc} C_{{p,{\text{sc}}}} \Delta T = 0.1274\,{\text{MJ}},$$
  14 $$E_{sc,heat - 2} = w_{sc} C_{{p,{\text{sc}}}} \Delta T = 0.4990\,{\text{MJ}}.$$

For the steam activation, the energy requirements are mainly for (i) heating the biochar from 500 to 700 °C (*E*_char,heat_), (ii) holding the biochar at 700 °C for 2 h (*E*_char,hold_) and (iii) producing steam (*E*_*boiler*_).

(i) Energy required to heat biochar was estimated from the sensible heat according to Eq. (15).
  15 $$E_{{{\text{char}},heat}} = w_{char} C_{{p,{\text{char}}}} \Delta T = 0.1013\,{\text{MJ}}.$$
(ii) Energy required for holding the biochar at 700 °C for 2 h was computed from the voltage–current–time profile of the furnace, which was equal to 40.3200 MW.
(iii) Energy required in boiler was estimated from the property of generated steam, which was at 1.2 barg. Based on the quantity of required steam, the amount of required energy for boiler was around 0.0016 MJ.

In summary, the total energy required to produce AC from spent disposable wooden chopsticks was the summation of the energy required for carbonization (11.7977 MJ/kg chopstick) and activation (40.4229 MJ/kg chopstick) with a total of 52.2206 MJ/kg chopstick. Therefore, the total energies required to produce AC and to adsorb CO_2_ based on the production yield of AC (23.18%) were 225.28 MJ/kg AC and 116.4 MJ/g-mol CO_2_, respectively. The estimated energy consumption was seemed to be high but was still in the range of biomass-derived AC, of 43.4 – 277 MJ/kg AC^81^.

## Conclusion

In this work, a series of ACs was prepared from spent disposable wooden chopsticks by steam activation for CO_2_ separation from a CO_2_/H_2_ mixed gas. From ANOVA analysis, it was found that a high activation temperature and long activation time (900 °C, 2 h) negatively affected the production yield and properties of ACs. From an economical point of view and adsorption performance, the optimal activation temperature and activation time for the preparation of AC was found to be 700 °C for 2 h, providing an experimental production yield of 23.18% and CO_2_ adsorption of 85.19 mg/g at 25 °C and 1 atm, respectively with the total energies required to produce AC carbon and to adsorb CO_2_ of 225.28 MJ/kg AC and 116.4 MJ/g-mol CO_2_, respectively. A fast breakthrough of H_2_ was observed via the as-prepared ACs over an inlet CO_2_ concentration in a mixed gas of 10–50 mol%, with the release of an almost pure H_2_ gas stream during the first 2 min of adsorption. The experimental data of CO_2_ adsorption was adequately described by the Freundlich isotherm model, where the physical adsorption played a predominate role on the interaction between the AC-CO_2_ molecules. The optimal AC exhibited a 18% loss of CO_2_ adsorption after six adsorption/desorption cycles. To further increase the CO_2_ absorption capacity, future research should be carried out to develop a large number of basic sites on the surface of AC such as adding basic metal oxides or alkali metals.

## Data Availability

All data generated or analyzed during this study are included in this published article.

## List of symbols

Δ*H*_vap_: Heat of vaporization of water (2.257 MJ/kg)^79^
*C*_*in*_: Inlet molar flow rate of CO_2_ (mmol/min)
*C*_*out*_: Outlet molar flow rate of CO_2_ (mmol/min)
*C*_*p*__,char_: Heat capacity of obtained biochar (J/kg K)
*C*_*p*__,__*sc*_: Heat capacity of chopstick (J/kg K)
*C*_*p,w*_: Heat capacity of chopstick (0.00421 MJ/kg K)
*E*: Mean free energy of adsorption (J/mol)
*k*_*F*_: Freundlich constant (mg/g atm^1^^/^^n^)
*k*_*L*_: Langmuir constant (atm^−^^1^)
*m*: Molar flow rate of gas (mol/min)
*M*: Composition of outlet gas stream (%)
*M*_*W*_: Molecular weight of CO_2_ (g/mol)
*n*: Freundlich exponential or adsorption intensity (–)
*N*: Number of data points (–)
*P*_*e*_: Equilibrium pressure of the CO_2_ adsorbed (atm)
*P*_*sat*_: Saturation pressure of CO_2_ at 25 °C (atm)
*q*: CO_2_ adsorption capacity of adsorbent (mg/g)
*q*_*D*_: Maximum adsorption of D–R model (mg/g)
*q*_*e*_: Equilibrium CO_2_ adsorption from experiment (mg/g)
$\overline{q}_{e}$: Average value of equilibrium CO_2_ adsorption from experiment (mg/g)
*q*_e,mod_: Equilibrium CO_2_ adsorption from model (mg/g)
*q*_*m*_: Maximum monolayer adsorption of CO_2_ (mg/g)
*R*: Universal gas constant (8.314 J/mol K)
*R*^2^: Determination coefficient (–)
*S*: Normalized standard deviation (%)
*S*_BET_: Total surface area (m^2^/g)
*S*_t__-__plot_: Micropore surface area (m^2^/g)
*t*: Adsorption time (min)
*T*: Absolute temperature (K)
*V*_mes_: Mesopore volume (cm^3^/g)
*V*_mic_: Micropore volume (cm^3^/g)
*w*: Amount of the adsorbent (g)
*w*_*AC*_: Weight of obtained activated carbon (g)
*w*_*m*_: Moisture content in chopstick (kg)
*w*_*SC*_: Weight of spent chopstick (g)
*Y*: Yield of activated carbon (%)

## References

[1] Hosseini S (2015). CO_2_ adsorption on modified carbon coated monolith: Effect of surface modification by using alkaline solutions. Appl. Surf. Sci..

[2] Udara Willhelm Abeydeera LH, Wadu Mesthrige J, Samarasinghalage TI (2019). Global research on carbon emissions: A scientometric review. Sustainability.

[3] Lamb WF (2021). A review of trends and drivers of greenhouse gas emissions by sector from 1990 to 2018. Environ. Res. Lett..

[4] Zhongming, Z., Linong, L., Wangqiang, Z. & Wei, L. The role of CCUS in low-carbon power systems. *International Energy Agency,* 49 pages (2020).

[5] Chou C, Chen F, Huang Y-J, Yang HJCET (2013). Carbon dioxide capture and hydrogen purification from synthesis gas by pressure swing adsorption. Chem. Eng. Trans..

[6] Saidi M, Gohari MH, Ramezani AT (2020). Hydrogen production from waste gasification followed by membrane filtration: A review. Environ. Chem. Lett..

[7] Casas N, Schell J, Pini R, Mazzotti M (2012). Fixed bed adsorption of CO_2_/H_2_ mixtures on activated carbon: Experiments and modeling. Adsorption.

[8] Nazir H (2020). Is the H2 economy realizable in the foreseeable future? Part III: H_2_ usage technologies, applications, and challenges and opportunities. Int. J. Hydrogen Energy.

[9] Idris, I., Abdullah, A., Shamsudin, I. & Othman, M. In *AIP Conference Proceedings.* 020059 (AIP Publishing LLC).

[10] Naksusuk S, Tangsathitkulchai C (2019). Carbon dioxide capture in a fixed bed of coconut shell activated carbon impregnated with sodium hydroxide: Effects of carbon pore texture and alkali loading. Eng. J..

[11] Song T, Liao J-M, Xiao J, Shen L-H (2015). Effect of micropore and mesopore structure on CO_2_ adsorption by activated carbons from biomass. New Carbon Mater..

[12] Sarmin S, Tarek BEM, Rengaraju B, Rezaul Karim KM, Ong HR, Abdullah H, Khan MMR (2020). Palm oil derived alkyd resin synthesis catalyzed by SrO/Sr(OH)_2_ nanoparticles. J. Crit. Rev..

[13] Wei H (2012). Granular bamboo-derived activated carbon for high CO_2_ adsorption: The dominant role of narrow micropores. Chemsuschem.

[14] Salituro A, Westwood A, Ross A, Brydson R (2020). Sustainable and regenerable alkali metal-containing carbons derived from seaweed for CO_2_ post-combustion capture. Sustain. Chem..

[15] Do D, Wang K (1998). A new model for the description of adsorption kinetics in heterogeneous activated carbon. Carbon.

[16] Danish M, Parthasarthy V, Al Mesfer MK (2021). CO_2_ capture by low-cost date pits-based activated carbon and silica gel. Materials.

[17] Ello AS, de Souza LK, Trokourey A, Jaroniec M (2013). Coconut shell-based microporous carbons for CO_2_ capture. Microporous Mesoporous Mater..

[18] Toprak A, Kopac T (2017). Carbon dioxide adsorption using high surface area activated carbons from local coals modified by KOH, NaOH and ZnCl_2_ agents. Int. J. Chem. React. Eng..

[19] Sarwar A (2021). Synthesis and characterization of biomass-derived surface-modified activated carbon for enhanced CO_2_ adsorption. J. CO2 Util..

[20] Bhatta LKG (2015). Investigation of CO_2_ adsorption on carbon material derived from *Mesua ferrea* L. seed cake. J. Environ. Chem. Eng..

[21] Zhao Z (2021). Water caltrop shell-derived nitrogen-doped porous carbons with high CO_2_ adsorption capacity. Biomass Bioenergy.

[22] Ma C (2022). Biomass derived nitrogen and sulfur co-doped porous carbons for efficient CO_2_ adsorption. Sep. Purif. Technol..

[23] Ali UFM (2018). Optimization study on preparation of amine functionalized sea mango (*Cerbera odollam*) activated carbon for Carbon Dioxide (CO_2_) adsorption. Combust. Sci. Technol..

[24] Hidayu A, Muda N (2016). Preparation and characterization of impregnated activated carbon from palm kernel shell and coconut shell for CO_2_ capture. Proc. Eng..

[25] Othman FEC (2021). Activated carbon nanofibers incorporated metal oxides for CO_2_ adsorption: Effects of different type of metal oxides. J. CO2 Util..

[26] Martinez ML, Torres MM, Guzman CA, Maestri D (2006). Preparation and characteristics of activated carbon from olive stones and walnut shells. Ind. Crops Prod..

[27] Tran HN, Chao H-P, You S-J (2018). Activated carbons from golden shower upon different chemical activation methods: Synthesis and characterizations. Adsorpt. Sci. Technol..

[28] Hu X, Radosz M, Cychosz KA, Thommes M (2011). CO_2_-filling capacity and selectivity of carbon nanopores: Synthesis, texture, and pore-size distribution from quenched-solid density functional theory (QSDFT). Environ. Sci. Technol..

[29] Zhou J, Luo A, Zhao Y (2018). Preparation and characterisation of activated carbon from waste tea by physical activation using steam. J. Air Waste Manag. Assoc..

[30] González A, Plaza M, Rubiera F, Pevida C (2013). Sustainable biomass-based carbon adsorbents for post-combustion CO_2_ capture. Chem. Eng. J..

[31] Rashidi NA, Yusup S (2017). A review on recent technological advancement in the activated carbon production from oil palm wastes. Chem. Eng. J..

[32] Plaza M (2009). Development of low-cost biomass-based adsorbents for postcombustion CO_2_ capture. Fuel.

[33] Zhang Y-J, Xing Z-J, Duan Z-K, Li M, Wang Y (2014). Effects of steam activation on the pore structure and surface chemistry of activated carbon derived from bamboo waste. Appl. Surf. Sci..

[34] Yahya, M. A. *et al.* In *AIP Conference Proceedings.* 030023 (AIP Publishing LLC).

[35] Li J (2019). Selective preparation of biomass-derived porous carbon with controllable pore sizes toward highly efficient CO_2_ capture. Chem. Eng. J..

[36] Rashidi NA, Yusup S, Borhan A, Loong LH (2014). Experimental and modelling studies of carbon dioxide adsorption by porous biomass derived activated carbon. Clean Technol. Environ. Policy.

[37] Ogungbenro AE, Quang DV, Al-Ali K, Abu-Zahra MR (2017). Activated carbon from date seeds for CO_2_ capture applications. Energy Proc..

[38] Quan C, Su R, Gao N (2020). Preparation of activated biomass carbon from pine sawdust for supercapacitor and CO_2_ capture. Int. J. Energy Res..

[39] Plaza M (2010). Developing almond shell-derived activated carbons as CO_2_ adsorbents. Sep. Purif. Technol..

[40] Balsamo M (2013). CO_2_ adsorption onto synthetic activated carbon: Kinetic, thermodynamic and regeneration studies. Sep. Purif. Technol..

[41] Jung S (2021). Hierarchical porous carbon beads for selective CO_2_ capture. J. CO2 Util..

[42] Song C (2020). Converting poly (ethylene terephthalate) waste into N-doped porous carbon as CO_2_ adsorbent and solar steam generator. Green Energy Environ..

[43] Kopac T (2021). Hydrogen storage characteristics of bio-based porous carbons of different origin: A comparative review. Int. J. Energy Res..

[44] Singh VK, Kumar EA (2016). Measurement and analysis of adsorption isotherms of CO_2_ on activated carbon. Appl. Therm. Eng..

[45] Inyinbor A, Adekola F, Olatunji GA (2016). Kinetics, isotherms and thermodynamic modeling of liquid phase adsorption of Rhodamine B dye onto *Raphia hookerie* fruit epicarp. Water Resour..

[46] Adelodun AA, Ngila JC, Kim D-G, Jo Y-M (2016). Isotherm, thermodynamic and kinetic studies of selective CO_2_ adsorption on chemically modified carbon surfaces. Aerosol. Air Qual. Res..

[47] Guarín Romero JR, Moreno-Piraján JC, Giraldo Gutierrez LJC (2018). Kinetic and equilibrium study of the adsorption of CO_2_ in ultramicropores of resorcinol-Formaldehyde aerogels obtained in acidic and basic medium. J. Carbon Res..

[48] Abunowara M (2020). Characterization of mukah-balingian and merit-pila coals before and after subcritical CO_2_ exposure using surface-area techniques. J. Environ. Eng..

[49] Raganati F, Alfe M, Gargiulo V, Chirone R, Ammendola P (2018). Isotherms and thermodynamics of CO_2_ adsorption on a novel carbon-magnetite composite sorbent. Chem. Eng. Res. Des..

[50] Phadungbut P (2022). Hunsom, Adsorptive purification of CO_2_/H_2_ gas mixtures of spent disposable wooden chopstick-derived activated carbon: Optimal synthesis condition. Separ. Purif. Technol..

[51] Cagnon BT, Py X, Guillot A, Stoeckli F (2003). The effect of the carbonization/activation procedure on the microporous texture of the subsequent chars and active carbons. Microporous Mesoporous Mater..

[52] Yang T, Lua AC (2003). Characteristics of activated carbons prepared from pistachio-nut shells by physical activation. J. Colloid Interface Sci..

[53] Acevedo S, Giraldo L, Moreno-Piraján JC (2020). Adsorption of CO_2_ on activatedcarbons prepared by chemical activation with cupricnitrate. ACS Omega.

[54] Sreńscek-Nazzal J (2016). Modification of commercial activated carbons for CO_2_ adsorption. Acta Phys. Polon. A..

[55] Zhao X (2018). Removal of Cr^6+^ ions from water by electrosorption on modified activated carbon fibre felt. R. Soc. Open Sci..

[56] Sing KS (1985). Reporting physisorption data for gas/solid systems with special reference to the determination of surface area and porosity (Recommendations 1984). Pure Appl. Chem..

[57] Zhang H (2020). Constructing hierarchical porous carbons with interconnected micro-mesopores for enhanced CO_2_ adsorption. Front. Chem..

[58] Khalil SH, Aroua MK, Daud WMAW (2012). Study on the improvement of the capacity of amine-impregnated commercial activated carbon beds for CO_2_ adsorbing. Chem. Eng. J..

[59] David E, Kopac J (2014). Activated carbons derived from residual biomass pyrolysis and their CO_2_ adsorption capacity. J. Anal. Appl. Pyrolysis.

[60] Shahkarami S, Dalai AK, Soltan J (2016). Enhanced CO_2_ adsorption using MgO-impregnated activated carbon: Impact of preparation techniques. Ind. Eng. Chem. Res..

[61] Firdaus RM, Desforges A, Emo M, Mohamed AR, Vigolo B (2021). Physical and chemical activation of graphene-derived porous nanomaterials for post-combustion carbon dioxide capture. Nanomaterials.

[62] Bouchelta C, Medjram MS, Bertrand O, Bellat J-P (2008). Preparation and characterization of activated carbon from date stones by physical activation with steam. J. Anal. Appl. Pyrolysis.

[63] Yang H, Yan R, Chen H, Lee DH, Zheng C (2007). Characteristics of hemicellulose, cellulose and lignin pyrolysis. Fuel.

[64] Petrovic B, Gorbounov M, Masoudi Soltani S (2020). Influence of surface modification on selective CO_2_ adsorption: A technical review on mechanisms and methods. Microporous Mesoporous Mater..

[65] Li W (2008). Effects of carbonization temperatures on characteristics of porosity in coconut shell chars and activated carbons derived from carbonized coconut shell chars. Ind. Crops Prod..

[66] Bouchelta C (2012). Effects of pyrolysis conditions on the porous structure development of date pits activated carbon. J. Anal. Appl. Pyrolysis.

[67] Fu K, Yue Q, Gao B, Sun Y, Zhu L (2013). Preparation, characterization and application of lignin-based activated carbon from black liquor lignin by steam activation. Chem. Eng. J..

[68] Tiwari D, Bhunia H, Bajpai PK (2019). Synthesis, characterization, adsorption and thermodynamic studies of pure and binary CO_2_–N_2_ mixtures on oxygen enriched nanostructured carbon adsorbents. Braz. J. Chem. Eng..

[69] Miyamoto M, Fujioka Y, Yogo K (2012). Pure silica CHA type zeolite for CO_2_ separation using pressure swing adsorption at high pressure. J. Mater. Chem..

[70] Peralta D (2012). Comparison of the behavior of metal–organic frameworks and zeolites for hydrocarbon separations. J. Am. Chem. Soc..

[71] Hu J, Sun T, Liu X, Guo Y, Wang S (2016). Separation of CH_4_/N_2_ mixtures in metal–organic frameworks with 1D micro-channels. RSC Adv..

[72] Abdeljaoued A, Relvas F, Mendes A (2018). Simulation and experimental results of a PSA process for production of hydrogen used in fuel cells. J. Environ. Chem. Eng..

[73] Belmabkhout Y, Sayari A (2009). Adsorption of CO_2_ from dry gases on MCM-41 silica at ambient temperature and high pressure. 2: Adsorption of CO_2_/N_2_, CO_2_/CH_4_ and CO_2_/H_2_ binary mixtures. Chem. Eng. Sci..

[74] Kubonova L, Obalová L, Vlach O, Troppová I, Kalousek J (2011). Modelling of NO adsorption in fixed bed on activated carbon. Chem. Process Eng..

[75] Ibrahim, H. G. & Al-Meshragi, M. A. In *CO*_*2*_* Sequestration* (IntechOpen, 2019).

[76] Dada A, Olalekan A, Olatunya A, Dada O (2012). Langmuir, Freundlich, Temkin and Dubinin–Radushkevich isotherms studies of equilibrium sorption of Zn^2+^ unto phosphoric acid modified rice husk. J. Appl. Chem..

[77] Li J, Hitch M (2015). Carbon dioxide sorption isotherm study on pristine and acid-treated olivine and its application in the vacuum swing adsorption process. Minerals.

[78] Yu C-H, Huang C-H, Tan C-S (2012). A review of CO_2_ capture by absorption and adsorption. Aerosol. Air Qual. Res..

[79] Maski, D., Darr, M. & Anex, R. In *2010 Pittsburgh, Pennsylvania, June 20–June 23, 2010.* 1 (American Society of Agricultural and Biological Engineers).

[80] Chen Y-H (2017). Production of a solid bio-fuel from waste bamboo chopsticks by torrefaction for cofiring with coal. J. Anal. Appl. Pyrolysis.

[81] Liao M, Kelley S, Yao Y (2019). Generating energy and greenhouse gas inventory data of activated carbon production using machine learning and kinetic based process simulation. ACS Sustain. Chem. Eng..

