# Author Correction: Valorization of spent disposable wooden chopstick as the CO_2_ adsorbent for a CO_2_/H_2_ mixed gas purification

**DOI:** 10.1038/s41598-022-15531-w

**Published:** 2022-07-05

**Authors:** Wanida Koo-amornpattana, Woranart Jonglertjunya, Poomiwat Phadungbut, Sakhon Ratchahat, Naphaphan Kunthakudee, Benjapon Chalermsinsuwan, Mali Hunsom

**Affiliations:** 1 Department of Chemical Engineering, Faculty of Engineering, Mahidol University, 25/25 Phuttamonthon 4 Road, Salaya, Phuttamonthon, Nakhon Pathom 73170 Thailand; 2 Department of Chemical Technology, Faculty of Science, Chulalongkorn University, 254 Phayathai Road, Pathumwan, Bangkok, 10330 Thailand; 3 Associate Fellow of Royal Society of Thailand (AFRST), Bangkok, 10300 Thailand

Correction to: *Scientific Reports* https://doi.org/10.1038/s41598-022-10197-w, published online 15 April 2022

The original version of this Article contained an error in the References. The authors omitted the below Reference, which is listed below as Reference 50.

50. Phadungbut, P. *et al.* Adsorptive purification of CO_2_/H_2_ gas mixtures of spent disposable wooden chopstick-derived activated carbon: Optimal synthesis condition. *Sep. Purif. Technol.* **291**, 120948 (2022).

As a result, in the caption of Figure 1 (a),

“Representative SEM images (left) and HRTEM images (right) of the (a) biochar, (b) AC7-2 and (c) AC9-2 samples.”

now reads:

“Representative SEM images (left) and HRTEM images (right) of the (a) biochar^50^, (b) AC7-2 and (c) AC9-2 samples.”

As a result of the changes, the References have been renumbered.

The original Article has been corrected.

